# Obesity: the greatest epidemic of the 21^st^ century?

**DOI:** 10.1590/S1516-31802011000500001

**Published:** 2011-09-01

**Affiliations:** I MD, PhD. Associate Professor in the Discipline of Thoracic Surgery, Hospital das Clínicas, Faculdade de Medicina da Universidade de São Paulo, São Paulo, Brazil.; II IMD. Trainee Physician in Tracheal Surgery and Therapeutic Respiratory Endoscopy, Discipline of Thoracic Surgery, Hospital das Clínicas, Faculdade de Medicina da Universidade de São Paulo, São Paulo, Brazil.; III MD. Attending Physician in the Pediatric Gastroenterology Service, Children's Institute, Hospital das Clínicas, Faculdade de Medicina da Universidade de São Paulo, São Paulo, Brazil

Obesity is a severe health problem worldwide.^1^ Around 1.6 billion adults (over the age of 15 years) are considered to be overweight (body mass index (BMI) between 25 and 30 kg/m^2^) and 400 million, obese (BMI ≥ 30 kg/m^2^).^1^ By 2015, it is expected that 2.3 billion individuals around the world will be overweight and 700 million will be obese.^1^

The United States is generally classified as one of the countries with the highest obesity rates in the world.^2^ The prevalence of obesity in the United States is indeed high, exceeding 30% for both sexes and in almost all age groups and ethnic groups.^2^ Even so, the proportion of obese individuals still seems to be gradually increasing.^3^ An analysis by the Centers for Disease Control and Prevention (CDC) suggests that there was an increase of 1.1% between 2007 and 2009, which would correspond approximately to another 2.4 million individuals considered obese.^3^ This has led to the projection that by 2050, almost all the population of the United States will be classified as overweight (BMI > 25 kg/m^2^) or obese (BMI ≥ 30 kg/m^2^).^3^

Individuals who are classified as overweight or obese present a higher risk of developing systemic arterial hypertension, dyslipidemia, type 2 diabetes and liver diseases, and have a higher mortality rate due to cardiovascular diseases.^3^ Moreover, obese mothers have a higher chance of complications during delivery.^1^ For the mother, these complications may include gestational diabetes, hypertensive diseases and eclampsia, thromboembolic events and infections.^1^ For the newborns of obese mothers, there are higher rates of difficulties during delivery, macrosomia, asthma, preterm birth and perinatal mortality.^4,5^

The large numbers of obese individuals in the worldwide population have major implications for healthcare costs. Public and private healthcare services in the United States together spend an estimated 147 billion dollars a year on obesity-related diseases.^6^

The situation becomes even more alarming when the pediatric age group is analyzed. The prevalence of obesity during childhood and adolescence is an important public health problem both in developed and in developing countries.^7^ A study by Freedman et al.,^8^ using data from the Bogalusa Heart Study demonstrated that 77% of the children presenting excess weight became obese in adulthood. In the United States, over the last three decades, the prevalence of excess weight among children has more than doubled, reaching 31.9% for overweight and 16.3% for obesity between the years of 2003 and 2006.^7,9^

Brazilians’ weights have also been increasing over recent years. Figures are available from the Family Budget Survey (Pesquisa de Orçamentos Familiares, POF) 2008-2009, which was conducted by the Brazilian Institute for Geography and Statistics (Instituto Brasileiro de Geografia e Estatística, IBGE), in partnership with the Ministry of Health.^10^ In 2008-2009, excess weight affected around half of Brazilian men and women. In relation to the prevalence of weight deficit, excess weight was 28 times more frequent among men and 13 times more frequent among women. The obesity rate was 12.5% among men (one quarter of the cases of excess weight) and 16.9% among women (one third). Both overweight and obesity increased in frequency up to the age range from 45 to 54 years among men, and up to the age range from 55 to 64 years among women, and thereafter declined. Excess weight was more evident among men with higher income (61.8%), while it varied little among women (45-49%) across all income groups. This survey also showed that there had been a continuous increase in excess weight and obesity among the population aged 20 years or over from 1974 to the time of the survey. The prevalence of excess weight among men almost tripled, from 18.5% in 1974-1975 to 50.1% in 2008-2009. Among women, the increase was less: from 28.7% to 48%. Moreover, obesity more than quadrupled among men, from 2.8% to 12.4% and more than doubled among women, from 8% to 16.9%.

Among children and adolescents, excess weight and obesity were found very frequently from the age of five years onwards, in all income groups and in all Brazilian regions. In 2008, excess weight affected 33.5% of children aged five to nine years; in this group, 16.6% of the boys were obese, while 11.8% of the girls were obese. Excess weight was more common in urban areas than in rural areas: 37.5% and 23.9% among boys and 33.9% and 24.6% among girls, respectively. The southeastern region stood out, since 40.3% of the boys and 38% of the girls in this age group presented overweight. The survey revealed that the number of children aged five to nine years with excess weight had leapt upwards over the 34-year period. In 2008-2009, 34.8% of the boys presented weights greater than the range considered healthy by the World Health Organization. In 1989, this proportion had been 15%, and it had been 10.9% in 1974-1975. A similar pattern was observed among the girls, with 8.6% in the 1970s, rising to 11.9% at the end of the 1980s and reaching 32% in 2008-2009.^10^

Excess weight is a serious health problem both in developed and in developing countries. It leads to immense costs for healthcare services and is a rapidly growing problem. The growing numbers of children and adolescents with excess weight and obesity are just as worrying as the obesity rates among the adult population. Intensive government programs to combat obesity are needed, especially for the pediatric age group, with stimulation towards exercise practice and improvement of diet, along with drug therapy and, in selected cases, surgical treatment.^11^
